# Grey matter volume in healthy and epileptic beagles using voxel-based morphometry – a pilot study

**DOI:** 10.1186/s12917-018-1373-8

**Published:** 2018-02-20

**Authors:** Lisa Frank, Matthias Lüpke, Draginja Kostic, Wolfgang Löscher, Andrea Tipold

**Affiliations:** 1 Department of Small Animal Medicine and Surgery, University of Veterinary Medicine, Hannover, Germany; 2 Department of General Radiology and Medical Physics, University of Veterinary Medicine, Hannover, Germany; 3 Department of Pharmacology, Toxicology and Pharmacy, University of Veterinary Medicine, Hannover, Germany

**Keywords:** Voxel-based morphometry, Dogs, Epilepsy, Grey matter, MRI

## Abstract

**Background:**

One of the most common chronic neurological disorders in dogs is idiopathic epilepsy (IE) diagnosed as epilepsy without structural changes in the brain. In the current study the hypothesis should be proven that subtle grey matter changes occur in epileptic dogs. Therefore, magnetic resonance (MR) images of one dog breed (Beagles) were used to obtain an approximately uniform brain shape. Local differences in grey matter volume (GMV) were compared between 5 healthy Beagles and 10 Beagles with spontaneously recurrent seizures (5 dogs with IE and 5 dogs with structural epilepsy (SE)), using voxel-based morphometry (VBM). T1W images of all dogs were prepared using Amira 6.3.0 for brain extraction, FSL 4.1.8 for registration and SPM12 for realignment. After creation of tissue probability maps of cerebrospinal fluid, grey and white matter from control images to segment all extracted brains, GM templates for each group were constructed to normalize brain images for parametric statistical analysis, which was achieved using SPM12.

**Results:**

Epileptic Beagles (IE and SE Beagles) displayed statistically significant reduced GMV in olfactory bulb, cingulate gyrus, hippocampus and cortex, especially in temporal and occipital lobes. Beagles with IE showed statistically significant decreased GMV in olfactory bulb, cortex of parietal and temporal lobe, hippocampus and cingulate gyrus, Beagles with SE mild statistically significant GMV reduction in temporal lobe (*p* < 0.05; family- wise error correction).

**Conclusion:**

These results suggest that, as reported in epileptic humans, focal reduction in GMV also occurs in epileptic dogs. Furthermore, the current study shows that VBM analysis represents an excellent method to detect GMV differences of the brain between a healthy dog group and dogs with epileptic syndrome, when MR images of one breed are used.

## Background

Dogs with epilepsy are frequent patients in veterinary practice [1]. Idiopathic epilepsy (IE) is one of the most common chronic neurological disorders in dogs, which is defined as a disorder of the brain characterized by spontaneous recurrent epileptic seizures of unknown, genetic or suspected genetic origin [1–3]. A genetic component was considered in several breeds [4], especially in Australian Shepherds [5], Beagles [6], Belgian Shepherds [7], Border Collies [8], English Springer Spaniels [9], Golden Retrievers [10], Keeshonds [11], Labrador Retrievers [12], Lagotto Romagnolos [13, 14], Viszlas [15] and others. Affected dogs mostly have their first epileptic seizure with an age of 6 months to 6 years, interictal general and neurological examinations are normal [16, 17]. Because IE is a diagnosis of exclusion, several diagnostic approaches have to be considered: TIER I: history, general and neurological examination, blood tests and urine analysis, TIER II: adding magnetic resonance imaging (MRI) and cerebrospinal fluid (CSF) analyses, TIER III: in addition electroencephalographic examinations [17]. Structural epilepsy (SE), caused by intracranial lesions like vascular damage, inflammation, trauma, anomalies or neoplasia of the brain, or reactive seizures are further reasons for the occurrence of seizures [1, 17]. For diagnostic purposes MRI of the brain is used to detect structural changes [1] resp. to diagnose IE by exclusion of such changes [18]. However, using new techniques such as volumetric studies in human epileptic patients, a reduced or increased volume of several grey matter (GM) structures could be detected using voxel- based morphometry (VBM) [19].

VBM is an automatically computational quantitative method to analyse MR images and detect differences in brain morphologies for instance in grey matter volume (GMV), white matter volume (WMV) or other structures between two groups of subjects [19]. After several pre-processing steps MR images are normalized to a consistent space for comparing each voxel of the same region in MR images of subjects by statistical analysis [19–21]. Resulting differences in brain volumes can be displayed in statistical parametric maps (SPM) [20]. Only few volumetric studies have been performed in veterinary medicine, since the diversity of head and brain shape between dog breeds [22] limit the application of VBM in dogs. In one volumetric study differences in the ratio of ventricular system volume to brain volume between dogs with IE and a healthy dog group were detected as preliminary results, while white matter (WM) volume to GMV ratio was not different between these groups [23]. In another study abnormalities of the hippocampus were seen in 12%, an unilateral atrophy of hippocampus in 48% of dogs with IE compared to healthy dogs by measuring the surface areas of the hippocampus in each MR image plane [24]. No significant differences in hippocampus volume between dogs with IE and controls were found by Milne et al. [25]. Only after the reduction of the 95% reference interval in the chosen analysis an atrophy of hippocampus in 18 of 74 dogs with IE could be shown [25].

In the current study the VBM method was chosen because of its sensitivity in detecting subtle structural alterations, which cannot be measured by conventional MRI [26]. Aim of this study was to examine whether GMV differences exist between healthy and epileptic Beagles. This study was conducted in only one dog breed to examine an approximately uniform brain shape. The hypothesis should be proven that epileptic Beagles have reduced GMV, especially in hippocampus and basal nuclei, as well as increased GMV in frontal regions of the brain.

## Methods

### Animals

Fifteen Beagles were included in this study. Five Beagles (2 male, 3 male-neutered) with an average age of 29.6 months and an average weight of 17 kg, property of the Department of Small Animal Medicine and Surgery of the University of Veterinary Medicine Hannover, represent the healthy control group (seizure-free up to 10 years of age). Their T1 weighted (T1W) MR images were already available from another animal experiment [27]. The patient group included 10 client-owned Beagles (2 female, 3 female-neutered, 3 male, 2 male-neutered) with an average age of 73.5 months and an average weight of 19.5 kg, presented between 2012 to 2017 at the Department of Small Animal Medicine and Surgery of the University of Veterinary Medicine Hannover with a history of recurrent generalized tonic-clonic seizures. No significant difference occurred between the age of controls and Beagles with IE (*p* = 0.07), however the age between controls and Beagles with SE differed significantly (*p* = 0.003). The weight was similar between controls and all epileptic Beagles (*p* = 0.30). All epileptic Beagles received comprehensive diagnostic workup based on the described TIER II level [17] on request of their owners. IE could be diagnosed in 5, structural changes (vascular, inflammatory, neoplasia) of the brain and thus SE in the remaining 5 Beagles (Table 1). In August 2017 all owners were called by phone to receive further information on seizure frequency resp. outcome of the disease. By telephone conference eight of ten owners could be reached.

**Table 1:** Overview of Beagles and volume measurements of GMV, WMV and WBV achieved by using SPM12

Beagle	Sex	Age (mo)	Weight (kg)	Group	GMV (ml)	WMV (ml)	WBV (ml)
C1	m	14	17.2	Con	45.2	31.7	76.9
C2	m	16	17	Con	44.9	36.0	80.9
C3	mn	65	15.5	Con	43.1	36.0	79.1
C4	mn	38	15.2	Con	42.8	39.3	82.1
C5	mn	15	20.5	Con	45.9	35.7	81.6
P1	f	109	13.2	SE	40.4	37.2	77.6
P2	fn	28	15.9	IE	47.7	36.0	83.7
P3	f	82	24	SE	35.2	43.2	78.4
P4	mn	45	19.7	IE	39.2	42.7	81.9
P5	fn	60	18	SE	48.7	34.2	82.9
P6	fn	104	14.8	IE	41.5	37.9	79.4
P7	m	53	15.2	IE	40.2	40.1	80.3
P8	mn	82	26.8	IE	28.9	50.9	79.8
P9	m	96	23	SE	45.6	34.9	80.5
P10	m	76	25	SE	48.2	25.6	73.8
				Average Con	44.4	35.7	80.1
				Average IE	39.5	41.9	81.4
				Average SE	43.6	35.0	78.6
				Average Epi	41.6	38.5	80.1

*C* control, *P* patient, *f* female, *m* male, *fn* female-neutered, *mn* male-neutered, *mo* months, *kg* kilogram, *Con* controls, *IE* idiopathic epilepsy; *SE* structural epilepsy, *Epi* epileptic, *GMV* grey matter volume, *WMV* white matter volume, *WBV* whole brain volume, *ml* milliliters

### Magnetic resonance imaging (MRI)

T1W three-dimensional (3D) turbo field echo (TFE) images of all fifteen Beagles were performed with a Phillips Achieva 3 Tesla MRI Scanner (Phillips Medical Systems, Eindhoven, The Netherlands) with a circular surface coil with a diameter of 11 cm. Examinations were performed under general anesthesia and artificial ventilation. For premedication either acepromazine (0.05 mg/10 kg BW IM) or diazepam (0.5 mg/kg BW IV) together with levomethadone (0.2–0.6 mg/kg BW IV) were used. Anesthesia was induced with propofol (2 mg/kg BW IV) and maintained with isoflurane in air and oxygen. For image acquisition, dogs were placed in dorsal recumbency and the following protocol parameters were used: a repetition time of 11.2 ms to 11.5 ms with an echo time of 5.2 ms to 5.3 ms, a slice thickness of 0.7 mm and a slice interval of 0.0 mm. The flip angle was 8° and the field of view (FOV) varied from 210 mm to 220 mm.

### Pre-processing (Fig. 1)

All fifteen T1W MR images were manually extracted using the software Amira 6.3.0 (FEI, part of Thermo Fisher Scientific, Hillsboro, Oregon, USA; costs: 5500US$) to receive 3D brain images. With the FSL software version 4.1.8 (FMRIB Software Library, the University of Oxford, https://fsl.fmrib.ox.ac.uk/fsl/fslwiki/FslInstallation; free download available) all fifteen extracted brain images were matched to the image of one control Beagle with a 12 parameter affine transformation kernel (*Registration*). Same software segmented the 5 extracted brain images of control Beagles in GM, WM and CSF (*Segmentation*). For this step, averaged intensity values of each tissue type in each brain image region read from a histogram produced by FSL 4.1.8 and displaying frequency of every single intensity value in a brain image region [28] were applied. To reach an automatical segmentation, tissue probability maps (TPMs) of GM, WM and CSF were created. Therefore all 5 extracted GM, WM and CSF images of control Beagles were realigned to each other (*Realignment*) and a template for each tissue type was generated by Dartel-Toolbox in the software SPM12 (Wellcome Department of Cognitive Neurology, London, UK, http://www.fil.ion.ucl.ac.uk/spm/software; free download available) that was implemented in MATLAB 9.2 R2017a (The MathWorks, Natick, Massachusetts, USA; costs: 500€/year). Templates were smoothed with a 2 mm FWHM (Full Width Half Maximum) Gaussian kernel (*Smoothing*). Resulting TPMs were used to segment all 15 extracted brain images automatically with SPM12 (*Segmentation*). By re-using Dartel-Toolbox in SPM12, 4 GM templates were created representing an average of extracted GM images: extracted GM images of the 5 control Beagles produced the standard template, further, 3 study specific templates emerged: one of the 5 extracted GM images from Beagles with IE, one of the 5 extracted GM images from Beagles with SE and one of all 10 extracted GM images from all epileptic Beagles.

**Fig. 1:**
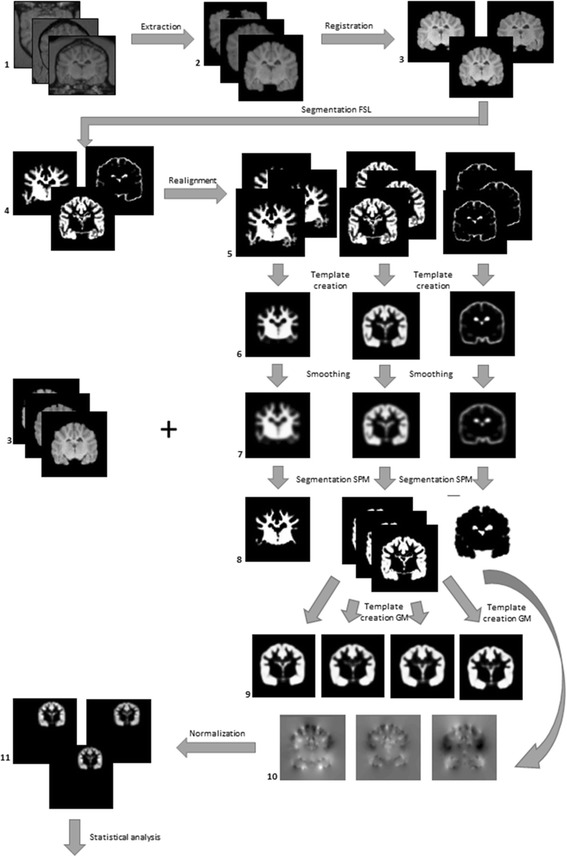
Overview of VBM pre-processing steps: 1: original T1W images; 2: extracted brain images; 3: registered brain images; 4: WM, GM, CSF images segmented by the software FSL; 5: realigned WM, GM, CSF images; 6: templates of WM, GM, CSF; 7: TPMs of WM, GM, CSF; 8: WM, GM, CSF images segmented by the Software SPM using TPMs; 9: template of control, IE, SE and epileptic Beagles; 10: flow fields; 11: normalized GM images (VBM: voxel- based Morphometry, T1W: T1 weighted, WM: white matter, GM: grey matter, CSF: cerebrospinal fluid, TPMs: tissue probability maps, IE: idiopathic epilepsy, SE: structural epilepsy)

To guarantee a voxel-wise comparison of GMV between groups, each extracted GM image was spatially normalized to an uniform space using flow fields by SPM12 (*Normalization*). These flow fields contain information of the necessary deformations by template creation and were created in this step. While normalization, smoothing of extracted GM images with a 2 mm FWHM Gaussian kernel was applied again as described by others [29].

### Statistical analysis using voxel-based morphometry

After the described steps extracted, registered, segmented, realigned, smoothed and spatially normalized GM images were taken for statistical analysis by using SPM12 [28–30]. Three VBM analyses were performed and a two-sample t-Test was chosen to compare GMV differences between groups. In analysis 1 control Beagles group were compared with IE Beagles group, in analysis 2 control Beagles group with SE Beagles group and in analysis 3 control Beagles group with epileptic Beagles group (IE and SE Beagles). Whole brain volume (sum of GMV and WMV) of each brain was taken as a covariant to correct for some large brain differences between study Beagles. Computation of brain volume (Table 1) was achieved by using the toolbox *tissue volume* in SPM12. In the option *Masking*, that pretends which voxel is included in the analysis [31], every voxel with a value of 0 (*implicit masking*) or a value below 0.01 (*threshold masking* with an *absolute threshold of 0.01)* was excluded from the analysis. The rest of settings in this step were adopted as required. The *classic method* was set in the *Estimate* option, in which the analysis is passed through. To show the *results* of t-statistics, two *t-contrasts* were defined: contrast a (1–1) defined that control Beagles group has larger contrast values than IE/SE/epileptic Beagles group, contrast b (− 1 1) defined that control Beagles group has less contrast values than IE/SE/epileptic Beagles group [30]. Furthermore, a *FWE* (family- wise error correction) for controlling chance of false positives [21] with a *p- value < 0.05* was used as well as a minimum limit for displaying clusters with at least 3 resp. 10 voxels (*extend threshold* set to *3* resp. *10).* Extend threshold of 3 voxels was chosen in order to minimize the occurrence of isolated randomly changed voxels in illustration of the VBM results, 10 voxels to represent larger GMV differences between groups that might have a clinical relevance. Therefore, all voxels of the GM that differed between groups with significance to a *p* value of < 0.05 and joined together in clusters with at least 3 resp. 10 voxels were shown as statistical parametric maps for instance in color maps [21].

## Results

The results of VBM statistics were displayed in statistical parametric maps in different views to identify the exact structures of GMV differing between groups of subjects. Specification of anatomical structures is based on definitions published by Uemura [32], Stoffel [33] and Palazzi [34].

### Group comparison

A comparison of the following groups using VBM was performed: controls and Beagles with IE, controls and Beagles with SE, controls and all epileptic Beagles, Beagles with IE and SE.

### Controls vs. IE

**Fig. 2:**
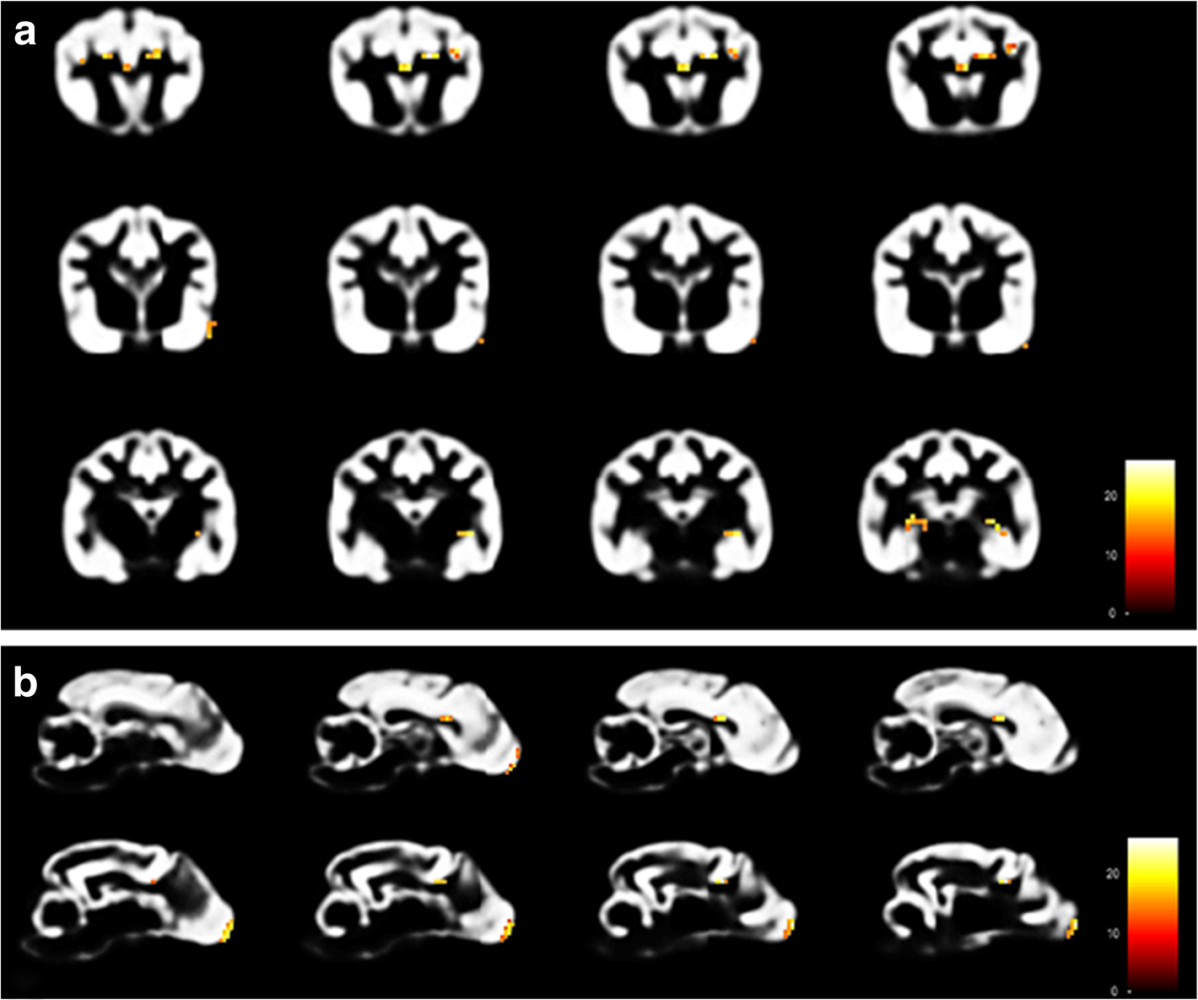
Results of VBM analysis 1 (control vs. IE Beagle group) using SPM12. Transversal [**a**] and Sagittal [**b**] view of parts of the GM-template of control Beagles illustrating regions of significant reduced GMV (coloured voxels) in the olfactory bulb, cingulate gyrus, cortex of parietal and temporal lobe and hippocampus in the IE Beagle group (VBM: voxel-based morphometry, IE: idiopathic epilepsy, GM: grey matter, GMV: grey matter volume)

**Fig. 3:**
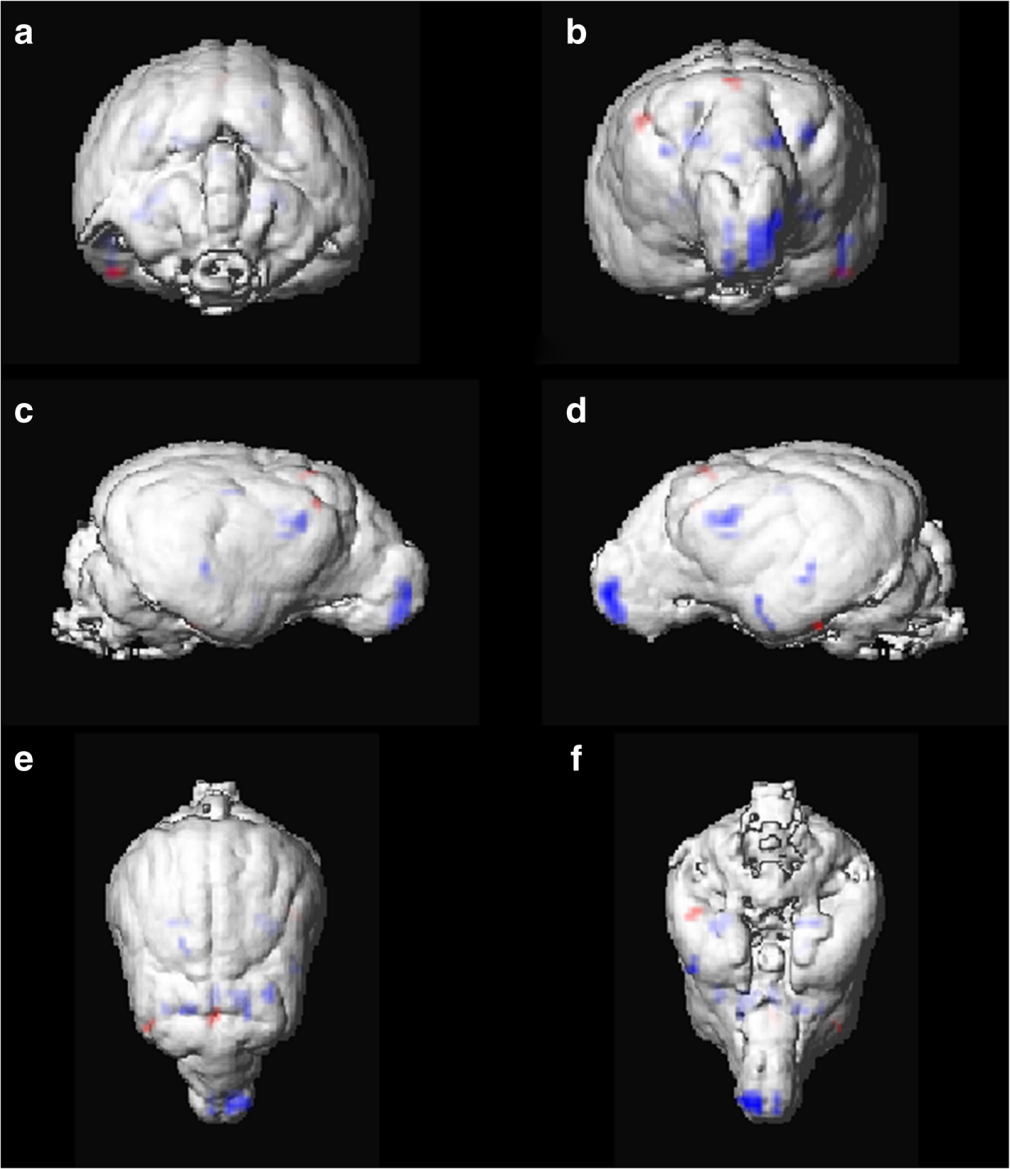
Results of VBM analysis 1 (control vs. IE Beagle group) using SPM12 illustrated on surface rendering of mean extracted GM images of control Beagles. Statistically significant reduced (blue) and increased (red) GMV is displayed in caudal [**a**], rostral [**b**], lateral right [**c**] and left side [**d**], dorsal [**e**] and ventral [**f**] views (VBM: voxel-based morphometry, IE: idiopathic epilepsy, GM: grey matter, GMV: grey matter volume)

When compared to controls using a threshold of FWE-corrected *p* < 0.05 and an extend threshold of 3 voxels, IE Beagles had statistically significant GMV reductions in the olfactory bulb bilaterally as well as in both cingulate gyri. Furtheron, statistically significant decreased GMV was found in the cortex of parietal (GM below coronal sulcus bilaterally and right marginal sulcus) and temporal lobe (left sylvian gyrus) and the hippocampus bilaterally (Table 2, Figs. 2 and 3). VBM comparison of IE group with control group also yielded statistically significant increased GMV in IE Beagles in the cortex of frontal (rostral part of right suprasylvian gyrus) and parietal lobe (precruciate gyrus near or in cruciate sulcus) as well as in the left piriform lobe (Table 3, Fig. 3). Setting the extend threshold to 10 voxels to show eventually clinical relevant aspects still displayed significant decreased GMV in IE Beagles in left olfactory bulb, left cingulate gyrus and left hippocampus compared to healthy Beagles (Table 2). Increased GMV regions did not reach this threshold.

**Table 2:** Regions of statistically significant reduced GMV in IE, SE and epileptic Beagles compared to healthy control Beagles

Major brain region	Region of reduced GMV	Voxel per cluster	Z score	PFWE-corr	Group
Olfactory	left olfactory bulb	34	5.54	0.000	IE
	right olfactory bulb	4/3	4.95/4.7	0.011/0.02	
Parietal	left cingulate gyrus	15	5.49	0.001	
	right cingulate gyrus	8	5.29	0.002	
	middle part cingulate gyrus	6	5.22	0.003	
	GM below left coronal sulcus	9	5.38	0.001	
	GM below right coronal sulcus	3	5.00	0.009	
	GM below right marginal sulcus	3	4.78	0.019	
Temporal	left sylvian gyrus	6	4.87	0.017	
	left hippocampus	10	5.23	0.003	
	right hippocampus	7	4.91	0.014	
Temporal	GM below left medial ectosylvian sulcus	3	5.03	0.008	SE
Occipital	left occipital gyrus	3	4.88	0.017	
Olfactory	left olfactory bulb	45	6.26	0.000	Epileptic
	right olfactory bulb	51	6.19	0.000	
Frontal	frontal gyrus concentral	31	5.90	0.000	
	right frontal gyrus	10	5.70	0.000	
Parietal	left cingulate gyrus	11/ 11	5.81/ 5.47	0.000/ 0.001	
	right cingulate gyrus	5/ 14	5.16/ 5.54	0.004/ 0.000	
	central cingulate gyrus	11	5.60	0.000	
	GM below left coronal sulcus	73	5.97	0.000	
	GM below right coronal sulcus	21	5.75	0.000	
Temporal	left putamen/claustrum	35	5.69	0.000	
	right putamen/claustrum	43	5.59	0.000	
	left sylvian gyrus	44/8	6.42/ 5.13	0.000/ 0.004	
	right sylvian gyrus	7	5.08	0.006	
	GM below left ectosylvian sulcus	53	6.21	0.000	
	GM below right ectosylvian sulcus	35	5.99	0.000	
	GM below left medial suprasylvian gyrus	3/3	4.81/ 4.72	0.012/ 0.012	
	GM below right medial suprasylvian gyrus	4	4.94	0.012	
	right suprasylvian gyrus	4	4.94	0.012	
	left hippocampus	67/13/6	5.76/ 5.99/ 5.32	0.000/ 0.000/ 0.002	
	right hippocampus	49/5/16	5.89/ 4.35/ 6.26	0.000/ 0.001/ 0.000	
	GM below left marginal sulcus	4	5.38	0.001	
	GM below right marginal sulcus	23	6.17	0.000	
Occipital	occipital gyrus	8/17/6/144	5.15/ 5.39/ 5.21/ 6.28	0.004/ 0.001/ 0.003/0.000	
	GM below medial suprasylvian gyrus	3/3	5.01/ 5.00	0.008/ 0.009	
Cerebellar	left cerebellum	6/6/6/7	5.85/ 5.53/ 5.37/ 5.09	0.000/ 0.000/ 0.001/ 0.005	
	right cerebellum	9/12/7/5	5.73/ 5.39/ 5.29/ 5.12	0.000/ 0.001/ 0.002/ 0.005	

Underlined: significant changes in GMV even with an extend threshold of 10 voxels (*GMV* grey matter volume, *GM* grey matter, *IE* idiopathic epilepsy, *SE* structural epilepsy)

**Table 3:** Regions of statistically significant increased GMV in IE, SE and epileptic Beagles compared to healthy control Beagles

Major brain region	Region of increased GMV	Voxel per cluster	Z score	PFWE-corr	Group
Frontal	right suprasylvian gyrus	3	5.05	0.007	IE
Parietal	precruciate gyrus/cruciate sulcus	4	5.11	0.005	
Temporal	left piriform lobe	4	5.04	0.007	
Parietal	region coronal sulcus	8	4.99	0.009	SE
Olfactory	right olfactory bulb	3	4.90	0.015	Epileptic
Temporal	left sylvian gyrus	14	5.28	0.002	
	right sylvian gyrus	8	5.00	0.009	
	left suprasylvian gyrus cranial	55	5.67	0.000	
	right suprasylvian gyrus cranial	19/*5*	5.39/ 4.99	0.001/0.009	
	left piriform lobe	42	5.92	0.000	
	right piriform lobe	11	5.76	0.000	

Underlined: significant changes in GMV even with an extend threshold of 10 voxels (GMV: grey matter volume, IE: idiopathic epilepsy, SE: structural epilepsy)

### Controls vs. SE

In Beagles with SE statistically significant decreased GMV could be seen in the cortex of the temporal lobe (GM below the left medial ectosylvian sulcus) and occipital lobe in comparison to controls with FWE-corrected *p*-value < 0.05 and extend threshold of 3 voxels (Table 2). In contrast the GMV was statistically significant increased in the cortex of parietal lobe (region beneath the middle part of the left coronal sulcus) (Table 3). Setting the extend threshold to 10 voxels for clinical relevant GMV changes displayed no significant differences in GMV between control and SE Beagles.

### Controls vs. epileptic

**Fig. 4:**
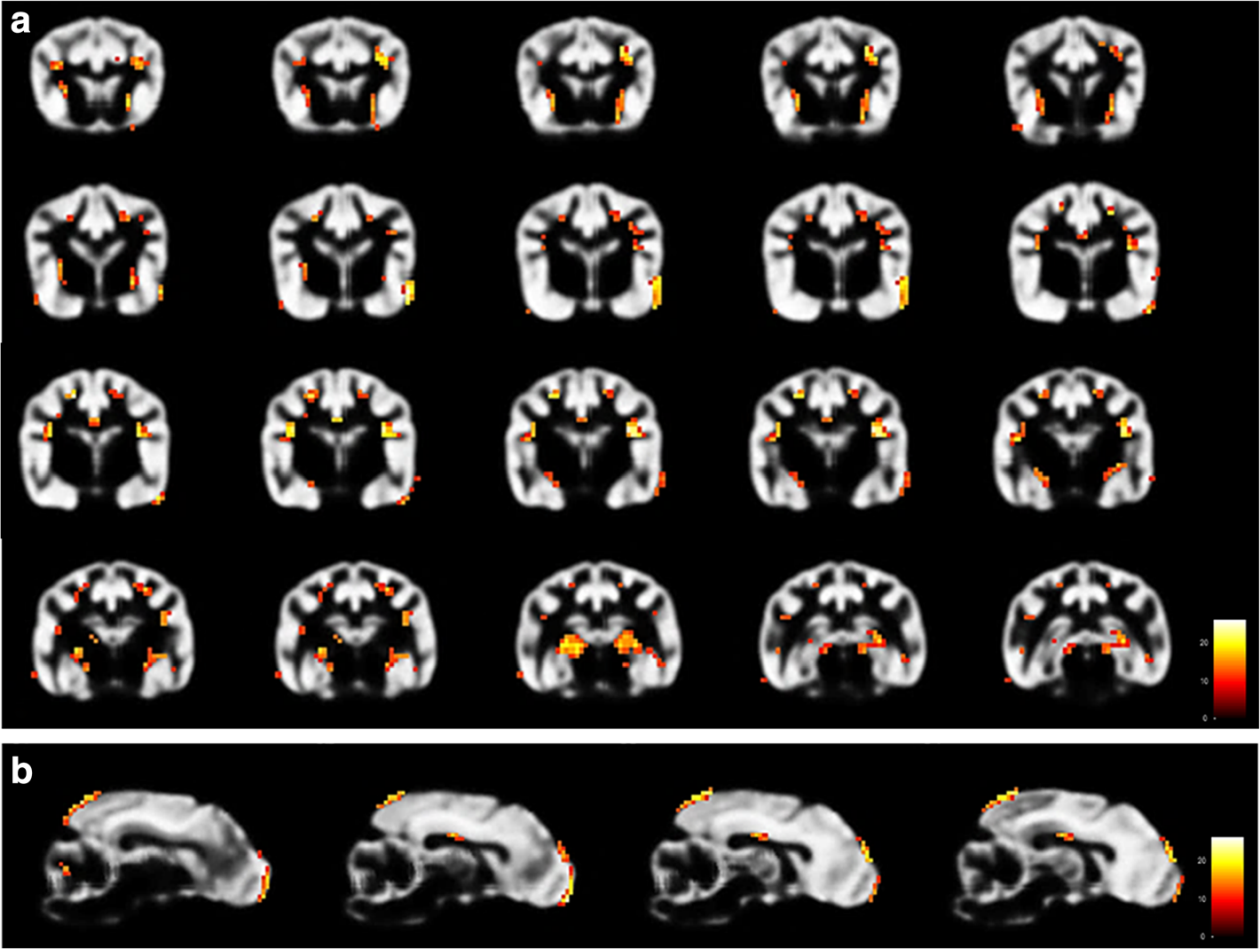
Results of VBM analysis 3 (control vs. epileptic Beagle group) using SPM12. Transversal [**a**] and Sagittal [**b**] view of parts of the GM-template of control Beagles illustrating regions of significant reduced GMV (coloured voxels) in the olfactory bulb, cortex of frontal, parietal, temporal and occipital lobe, cingulate gyrus, putamen, claustrum, hippocampus and cerebellum in the epileptic Beagle group (VBM: voxel-based morphometry, GM: grey matter, GMV: grey matter volume)

Comparing controls with all 10 epileptic Beagles (*p* < 0.05 FWE, extend threshold 3) several statistically significant changes of GMV in epileptic Beagles were detected. Regions with statistically significant decreased GMV were found in the olfactory bulb bilaterally, the cortex of frontal, parietal and temporal lobe. Further on, GMV reduction in epileptic Beagles was significant in both cingulate gyri and right and left putamen and claustrum (*p* < 0.05) (Table 2, Fig. 4). Epileptic Beagles also had decreased GMV in the hippocampus bilaterally, spacious regions of the occipital lobe and in cerebellum bilaterally in comparison to healthy Beagles (Table 2, Fig. 4). Statistically significant enhanced GMV in epileptic Beagles was found in the laterocaudal area of the right olfactory bulb, the cortex of temporal lobe and piriform lobe bilaterally (Table 3). Changing of extend threshold to 10 voxels emerged statistically significant reduced GMV in epileptic Beagles compared to controls in olfactory bulb, some parts of the cortex of frontal, parietal, temporal and occipital lobes, cingulate gyrus, putamen, claustrum and both hippocampi as before and mildly statistically significant decreased GMV in cerebellum on right side (Table 2). At this extend threshold of 10 voxels, epileptic group compared to control group displayed statistically significant increased GMV in temporal lobe (Table 3).

### IE vs. SE

No significant differences in GMV were detected between Beagles with IE and SE.

### Volume measurement and follow-up information by the owners

Six of ten epileptic Beagles showed reduced, four of ten increased whole GMV in comparison to controls (Table 1). Eight of ten owners were reached to evaluate the epileptic Beagles current condition and the outcome of the disease. Five of six Beagles with decreased whole GMV did not have a favorable response to antiepileptic drugs (AED) treatment; indeed, three were euthanized because of status epilepticus resp. no response to AED treatment on request of their owners. Two dogs with regular seizure activity despite treatment are still alive. Only one of six Beagles with reduced whole GMV became seizure free, but was euthanized because of another disease than epilepsy. In contrast, two of two Beagles with increased whole GMV are alive and seizure-free under appropriate medication.

## Discussion

Current pilot study was designed to examine grey matter volume differences between healthy control and epileptic dogs and to prove the feasibility of VBM methods in dogs. Results identified several brain regions with statistically significant reduced or increased GMV in Beagles with IE and SE compared to healthy control Beagles that are similar to in people. Most important significant volumetric differences were found in hippocampus, putamen/claustrum and cerebral cortex representing either the cause or consequence of epileptic seizures. Furthermore, in cingulate gyrus and frontal brain regions significant differences were detected that might explain behavioral changes in epileptic dogs.

VBM was chosen because of its sensitivity in detecting GMV differences between groups of healthy and epileptic humans, which cannot be seen in conventional MRI [19, 26]. Used MR images should have a relatively high resolution and be made with an at least 1 or 1.5 Tesla MRI Scanner to avoid preprocessing errors like inaccurate extraction or segmentation [20].

Using the VBM method not only specific structures of the brain can be evaluated, but also anatomical differences of the entire brain [19, 20]. Furthermore, VBM allows the assessment of atrophic brain regions across large groups of subjects without manual measurements and subjective influences [21]. However, the application of VBM method for animals, especially for dogs, has some disadvantages. Specialists for performing and evaluating VBM are necessary and the access of high performance computers is limited in veterinary medicine [22]. Variety of existing dog breeds resulting in brachycephalic, mesocephalic, dolichocephalic and mixed skull and brain shapes complicates standardized image processing and using VBM methods in canines routinely [22, 35]. In humans, skull shape with only minor variation in skull or brain size facilitates the application of VBM method. Customized brain templates, as they exist in human medicine, are rare or missing in veterinary medicine [22, 35]. Datta and others constructed a mesocephalic atlas of 15 brains of mesocephalic mixed-breed dogs [36], in another study a brachycephalic, mesocephalic and dolichocephalic as well as a mixed meso- and dolichocephalic skull shape specific template were created [22]. Two templates consisting of one dog breed, the Beagle [29], which is not publicly accessible, and the Dobermann Pinscher exist [37]. Such templates cannot be used for all dog breeds suffering from epilepsy. To overcome these difficulties, in the current study brain MR images of only one breed, the Beagle, were used to create a specific template for comparison analysis between healthy and epileptic brain MR images and to avoid analytical errors due to different brain shapes.

Application of VBM is widely used in human medicine in several neurological disorders [38–41], especially in epilepsy [42]. These studies detected subtle changes in brain volumes in epileptic humans [19, 26]. In contrast, VBM studies identifying structural brain changes in veterinary medicine are rare. Few studies have been performed in dogs to determine either brain volume in older dogs compared to younger ones [29], studying the influence of an antioxidant diet on cortical atrophy in aging dog brains [43] or structural brain abnormalities in canine compulsive disorders [37]. Other authors examined brain tissue volumes of dogs using volumetry [35] or studied the ventricular system to brain tissue volume ratio and WMV to GMV ratio, respectively [23], or hippocampal volume [44] between groups of healthy and epileptic dogs.

In the current study epileptic Beagles were examined. Described MR images of Beagle brains had to be manually extracted due to lack of a software that automatically extracts dog brains as accurately as manual extraction. Milne and others showed that manual brain extraction followed by the application of brain shape specific templates is the most accurate method for atlas-based segmentation in dogs [22]. Therefore, breed brain shape specific templates of GM, WM and CSF for a fully automatically brain segmentation was created [30, 31]. Smoothing with a 2 mm FWHM Gaussian kernel in the preprocessing step was chosen as described by others [29]. In order to minimize the occurrence of isolated randomly changed voxels in illustration of the VBM results the extend threshold of 3 voxels was chosen. In a second step only clusters with at least 10 altered voxels were displayed to represent large GMV differences between groups that might have clinical relevance.

Although VBM can detect differences in brain morphometry in smaller groups of subjects with expected large volume differences, greater power is achieved in groups with a high number of participants [20, 21, 45]. In the current pilot study MR images of 5 IE as well as 5 SE Beagles were used to evaluate the feasibility of the method for future studies with higher numbers of participants. Indeed, summarizing all 10 epileptic Beagles with spontaneous recurrent seizures, more frequent and larger GMV changes in statistical analysis were found in comparison to the evaluation of each group alone. Hence, VBM is a good method to detect brain volume changes between groups of subjects, but it cannot show reliable information for single-subject comparison [21]. VBM seems to be suitable for further epilepsy research in veterinary medicine evaluating certain groups of dogs with specific epileptic syndromes, but cannot improve the diagnostic workup of single epileptic patients.

Current VBM study revealed statistically significant reduced GMV in the frontal gyrus in epileptic dogs which is consistent with several previous studies made in human medicine. Reduced GMV was seen in some parts of the frontal lobe in patients with temporal lobe epilepsy (TLE) [46–50], juvenile myoclonic epilepsy (JME) [51], juvenile absence epilepsy (JAE) [52] and generalized tonic-clonic seizures (GTCS) [53] as well as frontal lobe epilepsy (FLE) [54]. Furthermore, statistically significant reduced GMV in several areas of the cortex of temporal and a few regions of parietal lobe in epileptic and IE Beagles and mildly statistically significant reduced GMV in the cortex of temporal lobe in SE Beagles was detected in the current study. Reduced GMV in cortices of temporal and parietal lobes are known in humans with TLE [48, 55]. Decreased GMV in areas of temporal lobe in TLE patients [45, 46, 56], in FLE patients [54] and patients with JAE [52] were also displayed in humans, while others found decreased GMV in parietal regions in brains of JME [57] or TLE patients [47, 49, 58]. Our study also revealed a statistically significant loss of GMV in occipital gyrus in SE and epileptic Beagles. These findings are consistent with several human medicine studies of TLE patients [46, 47, 49, 55, 58].

Many explanations for reduced GMV of the cerebral cortex in epileptic patients exist. Focal onset of epileptic seizures often spreads to the frontal lobe [59, 60] representing a pathway for interhemispheric propagation of ictal activity in humans with TLE [47]. In a family of Shetland Sheepdogs with IE Morita and others revealed an epileptic focus in the frontal lobe, which was consistent with acute neuronal necrosis [61]. Lateral TLE in humans seems to have a lateral temporal seizure onset [62]. Others hypothesize that hypometabolism in the temporal pole of humans with TLE may be responsible for GMV loss in this area [56]. Reduced GMV in temporal lobe cortex could even be involved in cognitive impairment in children with FLE [54]. In addition, the network of thalamus and the cerebral cortex might play a role in the mechanism of generalized seizures [42]. Reduced GMV in the cortex of frontal, parietal, temporal and occipital lobes in epileptic dogs might represent the onset focus of epileptic seizures, a lesion caused by epileptic seizures themselves or might be the consequence of hypometabolism in the affected regions. Behavioral changes occasionally observed in epileptic dogs [63] could also be the sequelae of decreased GMV in frontal parts of the brain as suggested by Woermann and colleagues in humans with TLE [58].

In contrast, increased GMV of frontal lobe areas was found in humans with JME [51, 57, 64, 65], TLE [47, 48], epileptic patients with hippocampal sclerosis [50] as well as FLE [54]. Keller and colleagues described additionally larger GMV in parietal cortex of human patients with TLE [47]. Even in epileptic humans with hippocampal sclerosis and TLE areas of increased GMV in temporal lobe could be detected [50, 58]. These results are consistent with findings of the current study. Mildly statistically significant increased GMV in the frontal lobe occurred in Beagles with IE compared to healthy controls as well as increased GMV in the cortex of parietal lobe in IE and SE Beagles. Furthermore, temporal lobe showed GM areas of statistically significant increased volume in epileptic Beagles. An increased grey matter volume could represent microdysgenesis and increased neuronal density [65–67] or reflect compensatory mechanisms such as increase in synapses because of reduced functional input to these areas [54]. Others suggest that higher frontal GMV in TLE patients before and after surgery with good outcome may reflect tissue recovery or protective mechanism for prevention of recurrent seizures [48]. A similar theory is that the cortex may play a leading role in seizure generation [57]. Higher GMV in cortex in epileptic Beagles may reflect tissue recovery or a compensatory mechanism because of reduced functional input in such brain areas. Furthermore, the cortex may play a leading role in seizure generation. Diminished GM to WM demarcation in temporal lobe as it is described in one human study in TLE patients [68] could be another explanation for our findings in epileptic Beagles.

Statistically significant reduced GMV in cingulate gyrus could be found in our study in epileptic dogs which is in accordance with several findings in human TLE [45, 46, 69] as well as in epileptic humans with JME and juvenile AE [52, 70]. On the other hand, in one study increased GMV in this part of the brain was described in patients with TLE compared to healthy controls [47]. In humans, it is known, that in mesial temporal lobe epilepsy seizures propagate to frontal lobe but also to cingulate areas [46]. This might explain GMV decrease in cingulate gyrus in described Beagles as a consequence of seizures themselves. In contrast, cingulate gyrus could also be considered as seizure focus as described in idiopathic generalized epilepsy (IGE) in humans [71]. Since cingulate gyrus plays a role in processing emotions [72], atrophic changes might explain postictal or general behavioral changes in epileptic dogs.

Lower GMV in putamen detected in the described Beagles was also shown in VBM analyses of human patients with neocortical seizures [73], IGE [74, 75] and TLE [45, 76]. Human patients with frontal, lateral temporal or occipital lobe seizures display a reduction in subcortical GMV including the putamen [73]. Because of connections between putamen and cerebral cortex [32], an abnormal cortex may result in fewer and aberrant dendrites projecting to the putamen and leading to GMV loss [75]. Since in the current study GMV loss was detected in cerebral cortex as well as in putamen, projection of dendrites damage is also feasible for epileptic Beagles. Furthermore, putamen atrophy may explain clinical symptoms like the tonic-clonic phase of generalized seizures [75].

The hippocampus of Beagles with IE had statistically significant reduced GMV that was even obvious, when all epileptic Beagles were calculated together. This finding is consistent with several human VBM studies about TLE [45, 46, 49, 55, 56, 69, 77]. Volumetric studies in veterinary medicine also showed hippocampal atrophy in epileptic dogs [24, 44]. Hippocampal sclerosis is the most common pathologic finding in human patients with mesial TLE [45, 78] and is suggested to be epileptogenic in patients with epilepsy [79]. Moreover, GMV loss in medial temporal lobe like hippocampus may also play a role in FLE and the etiology of cognitive impairment in epileptic patients [54]. Others suggest that damage of hippocampal neurons can also occur when the initializing seizure focus is located elsewhere [80]. Specific breed effects on hippocampal volume [24] and smaller hippocampal volumes in dogs of at least 11,5 years [81] were detected. Since the current study was performed in only one breed and the oldest epileptic Beagle was 9 years old, such physiological changes could be excluded and volume changes might be associated with epilepsy. Hippocampal atrophy is described as a consequence of seizures or status epilepticus in rats, mice and dogs [82–84] supporting the aforementioned assumption.

Current study even revealed statistically significant decreased GMV in cerebellum bilaterally. These findings are in accordance with results of several VBM studies of humans with TLE [45, 48, 49, 55, 69, 85], JME [51, 57] as well as GTCS [53]. The cerebellum has connections with a large number of other brain regions, especially the cerebral cortex [51, 86, 87] leading to the hypothesis that nerve cell injury and discharges spread to cerebellum and cause Purkinje cell loss [87–89]. Moreover, cerebellar hypometabolism is detected in patients with focal seizures [90]. Whereas the cerebellum seems to have an inhibitory effect on seizures, cerebellar lesions could lead to generation and spread of epileptic seizures [87, 90].

In epileptic Beagles GM volume changes of the olfactory bulb were seen, but not further considered. Different sizes and shapes of each individual olfactory bulb were noticed during manual extraction of MR images and might represent normal variations of the brain.

Additionally, GM and WM volumes of all 15 Beagle brains were determined using *tissue volume tool* in SPM12 and compared with volumetric investigations by others [23, 35]. WMV of epileptic Beagles was higher than volumes measured by Schulze et al. [35]. Since control Beagles had similar WMV as the different breeds used in the other volumetric study [35], an increased WMV in Beagles with IE could be the consequence of the described partly reduction of GMV. This hypothesis is supported by changed GMV to WMV as well as GMV to WBV ratios in IE and epileptic Beagles of the current study in comparison to these ratios computed by Schulze and colleagues [35], while it contradicts the assumption that dogs with IE have reduced WBV with preservation of WMV to GMV ratio [23].

In the current study five of six epileptic Beagles with reduced whole GMV than controls showed an unfavorable outcome because of their unresponsiveness to antiepileptic therapy, whereas two of two Beagles with increased whole GMV are currently seizure-free under medication. These preliminary findings are supported by results of a human study that showed that TLE patients with significantly reduced GMV in the frontal lobe, pre-surgery, will more likely experience a poor seizure outcome, post-surgery, while patients with higher GMV in frontal lobe pre-surgery, experience seizure freedom after surgery more frequently [48]. Therefore we suggest from our study that a loss of whole GMV in epileptic Beagles is associated with a poorer outcome than higher whole grey matter volumes. VBM could therefore be further evaluated as a biomarker for treatment outcome.

## Conclusion

In conclusion, in the current study the hypothesis was confirmed that epileptic Beagles have reduced GMV, especially in hippocampus and basal nuclei, as well as increased GMV in frontal regions of the brain. Additionally, further GMV differences between epileptic and healthy Beagles could be detected. All findings of volumetric differences between epileptic and healthy Beagles are in agreement with several findings in epileptic humans that emphasizes that the dog is a useful large animal model for humans [91–93]. Found reduction of grey matter volume in epileptic Beagles may represent the cause or consequence of epileptic seizures as well as be responsible for behavior changes of epileptic dogs, while an increased volume of grey matter can denote tissue regeneration or protective mechanisms. In this pilot study, it could be shown that VBM analysis represents an excellent method to detect GMV differences of the brain between groups of healthy and epileptic dogs, when MRI images of only one breed are compared. VBM cannot be recommended for evaluation of individual patients. Nevertheless, this technique has to be further developed in order to apply it broadly in veterinary medicine research.

## Abbreviations

AED: Antiepileptic drugs
CSF: Cerebrospinal fluid
FWE: Family-wise error correction
FLE: Frontal lobe epilepsy
GM: Grey matter
GMV: Grey matter volume
GTCS: Generalized tonic-clonic seizures
IE: Idiopathic epilepsy
IGE: Idiopathic generalized epilepsy
JAE: Juvenile absence epilepsy
JME: Juvenile myoclonic epilepsy
MR: Magnetic resonance
MRI: Magnetic resonance imaging
SE: Structural epilepsy
T1W: T1-weighted
TFE: Turbo field echo
TLE: Temporal lobe epilepsy
TPM(s): Tissue probability map(s)
VBM: Voxel-based morphometry
WBV: Whole brain volume
WM: White matter
WMV: white matter volume
